# Current News and Opinion

**Published:** 1886-12

**Authors:** 


### Current News and Opinion.

### FIRST DISTRICT DENTAL SOCIETY OF THE STATE OF NEW YORK.

A special meeting of this society was held in New York, November 19th. This was the largest and most enthusiastic meeting of that society which was ever held, the audience numbering nearly two hundred. It was called to hear Dr. Kingsley read a paper entitled "Dentistry Not a Specialty in Medicine." This address was, in substance, the same as that by the same author, delivered before the New England Society in October, and which will appear in THE INDEPENDENT PRACTITIONER for January. At the close of the reading the sentiments advanced were warmly endorsed by Drs. Parmly Brown, Atkinson, Rich, and Dwinelle.

The following resolutions were offered by Dr. Wm. H. Dwinelle, seconded by Dr. S. G. Perry, and adopted without a dissenting voice :

WHEREAS, Dentistry in America is practically an independent profession and not subordinate to any other, and—

WHEREAS, All the greatest attainments in dental science have been reached through separate literary; educational, and scientific organization, and—

WHEREAS, Dentists throughout the world look to their professional confreres in America for the further advancement of dental science; therefore,

*Resolved*, That in the interests of dentistry as an independent profession, immediate steps be taken looking to the formation of an INTERNATIONAL DENTAL CONGRESS, to be held in this country, and to which every reputable dentist in the world shall be entitled to admission and to all its privileges.

*Resolved*, That a committee of ten be appointed by the President, whose duty shall be to co-operate with similar committees from other societies, for the purpose of establishing such a Congress at as early a date as arrangements can be made which will make such a Congress a credit to the dental profession in America.

*Resolved*, That we respectfully recommend that every dental society in the world appoint a similar committee, and thus bring about harmonious relations and universal support.

The President subsequently appointed as members of the committee of ten, Drs. W. H. Dwinelle, A. L. Northrop, C. E. Francis, Frank Abbott, W. H. Atkinson, N. W. Kingsley, S. G. Perry, E. A. Bogue, W. A. Bronson and W. W. Walker.

At a union meeting of the Fifth, Sixth, Seventh and Eighth District Dental Societies, held in Rochester, October 26th and 27th, a paper was read by Dr. C. T. Howard upon the Relations of Dentistry to Medicine, and Dr. Kingsley presented the paper which was subsequently read before the First District Society.

Resolutions substantially the same as those passed by the First District Society were offered and unanimously adopted.

---

### BALD AND TOOTHLESS.

In a facetious leading article, called forth by the assertion of Mr. Virgil G. Eaton and Dr. William A. Hammond that our descendants of a thousand years hence will be destitute of hair and teeth, the *Journal of the American Medical Association* says :

Will this be an unmitigated evil to posterity? The dental college will then live only in history, and the barber, like Othello, will find that his "occupation's gone." The medical literature of that day will perhaps contain a short reference to a disease mentioned in the infant history of medicine as "Toothache," and doubtless some medico-historian will show that its disappearance from the earth was caused by a change in the meteorological conditions of the planet in the year 2900. The Professor of the History of Medicine will no doubt lecture learnedly on the fatal affection of the nineteenth century, known as "dentition," and will ascribe its disappearance to improvements in sewer pipes and house drainage. He will look up *alopecia* in the medical dictionary, and finding that it is so called because foxes were supposed to be afflicted with it, will marvel greatly that even so late as the twentieth century the doctor could not cure fleas. The archæologist will perhaps find an old toothbrush, and straightway construct the wondrous animal which possessed such a tail. And what a very treasure it will be to him when, in excavating the site of an ancient barber shop, he finds a bottle with the curious inscription : "Scalpine: positive cure for baldness." Perhaps some daring scientist will try some of it on a square centimeter of his own glossy scalp, and regret it for the remainder of his days. False teeth, teething rings and hairpins will be deposited in honorable positions in museums. No doubt Paracelsus will then get the credit of having invented the dental engine, and given it a name which, like many others of the nineteenth century, has no signification, and no other merit than length. In that day an infant king of Spain will not have a dentist appointed for him before he has taken leave of the last three inches of his umbilical cord. Roast beef will be a thing of the past, and the earth will be dotted over with mush factories.—*Practitioner and News.*

---

### CONNECTICUT VALLEY DENTAL SOCIETY.

At the annual meeting of the Connecticut Valley Dental Society, held at Holyoke, Mass., October 14th and 15th, the following officers were elected for the ensuing year :

*President*—E. A. Stebbins, Shelburne Falls, Mass.

*Vice-Presidents*—J. N. Davenport, Northampton, Mass.; F. W. Williams, Greenfield, Mass.

*Secretary*—George A. Maxfield, Holyoke, Mass.

*Assistant Secretary*—A. J. Nims, Turners Falls, Mass

*Treasurer*—W. H. Jones, Northampton, Mass.

The semi-annual meeting for 1887 will be held at Montreal, P. Q., next June, and a cordial invitation is extended to members of the profession to attend. Special arrangements will be made with railroads and hotels for reduced rates, and an attractive programme will be provided. Additional notice will be given as early as next April.

GEO. A. MAXFIELD, D. D. S.,

Secretary.

HOLYOKE, Nov. 1, 1886.

### AMERICAN ACADEMY OF DENTAL SCIENCE.

The annual meeting of this society was held at Young's Hotel, in Boston, Wednesday, November 10th The annual address was delivered by J. N. Farrar, M. D., D. D. S., of New York. (It will be published in full in THE INDEPENDENT PRACTITIONER.—ED.)

The officers of the preceding year were all re-elected, as follows :

*President*—Dr. J. H. Batchelder.

*Vice-President*—Dr. C. P. Wilson.

*Recording Secretary*—Dr. E. E. Hopkins.

*Corresponding Secretary*—Dr. E. B. Hitchcock.

*Treasurer*—Dr. E. H. Smith.

*Librarian*—Dr. H. C. Merriam.

*Committee of Arrangements*—Dr. C. P. Wilson, Dr. E. C. Briggs, Dr. J. S. Mason.

---

### NEW ENGLAND DENTAL SOCIETY.

The officers elected at the last meeting of this society were :

*President*—Dr. G. A. Young, Concord, N. H.

*First Vice-President*—Dr. C. W. Clement, Manchester N. H.

*Second Vice-President*—Dr. C. A. Brackett, Newport, R. I.

*Secretary*—Dr. A. M. Dudley, Salem, Mass.

*Assistant Secretary*—Dr. A. H. Gilson, Boston, Mass.

*Treasurer*—Dr. G. A. Gerry, Lowell, Mass.

*Librarian*—Dr. E. O. Kinsman, Cambridge, Mass.

*Executive Committee*—Dr. A. M. Dudley, Dr. L. D. Shepard, Dr. R. R. Andrews, Dr. H. A. Baker, Dr. Geo. C. Ainsworth.

The next meeting, at Boston, will be the 25th anniversary and a big meeting, occurring in October, 1887.

---

### "THE OLD RELIABLE."

Ambrose Lawrence, M. D., originator of the famous "Lawrence's Amalgam," has made The S. S. White Co. agents for the sale of his filling, while he devotes his attention solely to its production.

Many years ago the venerable doctor practiced dentistry in Lowell, Mass., and for a time was mayor of that thriving city; but the demand for his "stuffing" became so great that he gave up dental practice, migrated to Boston, and became one of the spokes of the "Hub," making his revolutions in regular time and order.

The doctor, like his amalgam, is—well, *somewhat* "old," but exceedingly "reliable," and we are pleased to learn that he is as hale and robust as any other "boy" of his size that can be brought to the front.

Long may he live and prosper, and in his battles with other amalgams may he show his sterling metal, and, while he lives, "never give up the ship."

C. E. F.

### THE ODONTOLOGICAL SOCIETY OF WESTERN PENNSYLVANIA.

This society will hold its next regular quarterly meeting at the office of Drs. Libbey, corner of Sixth Avenue and Smithfield Street, Pittsburgh, on Tuesday, December 14th next.

PROGRAMME.

ESSAY—"Odontalgia and its Treatment." By Dr. L. Depuy. Discussion opened by Dr. Geo. Elliott.

ESSAY—"Recent Advances in Dental Art." By Dr. H. W. Arthur. Discussion opened by Dr. J. A. Libbey.

Essay—"Abscesses and Their Treatment." By Dr. J. W. Boisol. Discussion opened by Dr. Sophy Feltwell.

There will also be an operative clinic, if time permits.

C. V. KRATZER, Secretary,

### CAULK'S DENTAL ANNUAL.

For statistics of dentistry the members of the profession have learned to consult this excellent compilation. Number V, to be published January 1, 1887, will be of more than usual interest and value, while the edition will be larger than that of any of its predecessors. Address Dr. L. D. Caulk, Camden, Del.

ACCORDING TO THE *Iowa State Medical Reporter*, diplomas from the following list of colleges will not be accepted by the Iowa State Board of Health, recently organized:

American Eclectic College, Cincinnati; American Health College, Cincinnati; American University of Pennsylvania (Buchanan), Philadelphia; Beach Medical Institute, Indianapolis; Bellevue Medical College, of Massachusetts; College of Physicians and Surgeons, Buffalo; College of Physicians and Surgeons, Milwaukee; Eclectic Medical College, of Philadelphia; Edinburg University, Chicago and St. Louis; Excelsior Medical College, Boston; Hygeo-Therapeutic College, Bergen Heights, N. J.; Joplin Medical College, Joplin, Mo.; Livingston University, Haddenfield, N. J.; Medical Department of the American University of Boston, Boston; New England University of Arts and Sciences, Manchester, N. H.; Penn Medical University, Philadelphia; Philadelphia University of Medicine and Surgery; Physio-Eclectic Medical, and Physio-Medical College, Cincinnati; St. Louis Eclectic Medical College; St. Louis Homœopathic Medical College; Physio-Medical Institute, Marion, Ind ; American Anthropological University, of St. Louis; Medical Department of Drake University, Des Moines; and King Eclectic Medical College, Des Moines, Iowa.

—*Kansas City Medical Index.*

IF THE SIMPLICITY OF PRAYER be its chief merit, then a colored preacher, who offered up a supplication at the Citadel Green on the night of the great earthquake, is far ahead of many of the courtly and polished theologians of the day. The following prayer, which is by many degrees more eloquent than the prayer of Ajax for light, was made before a terrified crowd of colored and white people on the Green on the night stated, and, considering the circumstances and the peculiar notions of the colored people on such matters, the preacher will be acquitted of anything like a charge of irreverence. He said, raising his hands towards the sky:

"Do, Lawd, come down an' go 'mong yer people, for dey is terrify—dey am skeerd like; dey dunno which way to tun over; 'cause dey tink you am bex wid um. Come down, please, sa, an' walk roun' wid um; tek um by de han' an' tell um you ain' bex; an' if yo' can't come, sen' you' Son. *But*, Lawd, *dis no time fo' chillun.* So come, yo'self, if you can, please, sa!"

The above is a reproduction of the prayer just as it was delivered, without an attempt at amplification or interpolation. It is easy to see how the colored divine, deriving his knowledge of the character and power of the Saviour from pictures in which he is represented as an infant, should have said exactly what he did say. The preacher's idea was that the case required the immediate attention of what he conceived to be the only and supreme authority.—*Charleston News.*

---

Dr. A. D. McGregor speaks highly of boric acid as a topical application in the unhealthy condition in which we frequently find the mouth, tongue and teeth in severe cases of typhoid fever. He says, in *The British Medical Journal:* The mouth is hot, the lips dry, cracked, and glued to the sordes-covered teeth by inspissated mucous and saliva; the tongue dry, or even glazed and hard, brown or black, crusted, with a fetid fur. Under these circumstances, a pigment containing boric acid (30 grains), chlorate of potassium (20 grains), lemon juice (5 fluid drachms), and glycerine (3 fluid drachms), yields very comforting results. When the teeth are well rubbed with this, the sordes quickly and easily becomes detached; little harm will follow from the acid present. The boric attacks the masses of bacilli and bacteria, the chlorate of potassium cools and soothes the mucous membrane, the glycerine and lemon juice moisten the parts and aid the salivary secretion.—*Medical Record.*

---

The Florence Manufacturing Co., as may be learned by a reference to their advertisement, have prepared for the holiday trade one of the neatest articles in the market. It consists of an ornamental case containing a brush, comb and glass, which would make an acceptable present to any one, and is especially adapted to office use. Few can realize the favorable impression made upon patients by an elegant attention to their minor wants, and if one of these sets were laid upon the dressing case of the office, it could not but be pleasing to any lady patient.

---

The Central Dental Association of Northern New Jersey holds its regular meeting on the third Monday of each month, at the rooms of the Board of Trade, Newark. All regular dentists are cordially invited to be present at any time. It is expected that the December meeting will be addressed by Dr. W. C. Barrett, of Buffalo; that of January, by Dr. A. W. Harlan, of Chicago.

S. C. G. Watkins, Chm'n Ex. Com.

Harvard University has just conferred the honorary degree of LL. D. upon Dr. J. S. Billings and Dr. S. Weir Mitchell. The high compliment thus paid to Dr. Billings is a just recognition of his eminent services to the science and literature of his profession, and a fitting rebuke to those gentlemen of the medical press of the west and south-west who have attempted to belittle Dr. Billings' fair name and fame by cheap criticisms and vulgar aspersions. We congratulate Dr. Billings upon the reception of this well-merited honor at this time from the oldest and most eminent University of the United States.—*Maryland Medical Journal.*

To all of which we most heartily subscribe.

---

Myra and Belfanti (*Centralt. f. Klin. Med.*, 1886, *No.* 26) have succeeded in detecting two digestive ferments in normal human urine. One is the already well-known digestive ferment, which is active in an acid solution ; the other displays its activity in an alkaline solution only. Both ferments produce only small quantities of peptone. The first ferment is found also in pathological conditions, typhoid fever, gastric cancer and Bright's disease. The ferments have nothing whatever to do with the putrefactive processes. The detection of these ferments, the authors believe, is of considerable importance in the question of the pathological significance of peptonuria or propeptonuria—*New York Medical Journal.*

---

Dr. Geo. L. Field, of Detroit, has for years been using a little device that has excited the envy of metal workers. It is a machine for turning the rims of gold and platina plates, and it does its work much better than it can be done by hand, and that, too, without any danger of injuring the fit At the solicitation of some of those who had seen it, he has finally had a few made from his patterns for those who especially ordered them, and it is possible that he could get more if they were desired. The one belonging to the editor of this journal will certainly never be for sale as long as he is able to use it.

---

The particular office of flies appears to be the consumption of those dead minute animals whose decaying myriads would otherwise poison the air. It was a remark of Linnæus that three flies would consume a dead horse sooner than a lion could. He, of course, included the families of the three flies. A single fly, *The Naturalist* tells us, will sometimes produce 20,000 larvæ, each of which, in a few days, may be the parent of another 20,000, and thus the descendants of these flies would soon devour an animal much larger than a horse.—*Sci. Am.*

---

In Austria and Hungary there are no dentists, medical men alone being allowed to practice dentistry. Before the year 1876, in order to practice dentistry in Holland it was necessary to hold the diploma of medicine, surgery and midwifery. The law has now been amended, a special course of study and special examinations having been provided for, as in Germany, Russia and Switzerland.

---

Pulque is the stimulant of Mexico. It is made out of the juice of the cactus, and is sold at a cent a glass. It is said to look bitter, smell loud, and taste yellow, but it gets there just the same.

Dr J. G. Van Marter, of Rome, Italy, has lately received a number of distinguished honors. His *Alma Mater*, Williams College, has conferred upon him the degree of Master of Arts. The Imperial German Archæological Society of Berlin, Rome and Athens has elected him a corresponding member, and the Vatican has sent him a silver medal for his literary and scientific work, no inconsiderable portion of which was given to the world through The Independent Practitioner. We hope, in the not distant future, to have another important article from him on archæological dentistry.

---

Dr. G. W. Melotte, of Ithaca, exhibited, at the last meeting of the American Dental Association, his method of getting an exact articulation in artificial crown and bridge-work, by reproduction of the precise contour of any tooth. It is done by means of an impression compound and fusible metal, and five minutes suffice to make a complete die for striking up the crown. Those who have seen it in use by Dr. Melotte will be glad to know that the material is now on sale by the S. S. White Manufacturing Co., who send complete instructions for use with every package.

---

Paul Bert, the well-known French physiologist and scientist, died at Paris, Nov. 11th. For the last ten years he had mainly devoted himself to political affairs, and hence his name was not as frequently seen in scientific journals as formerly. Dentists are greatly indebted to him for his researches upon anæsthetics, especially Nitrous Oxide Gas.

---

At a recent meeting of the Dresden Agricultural Society, a local pharmacist reported that, in the neighborhood where the deadly night-shade grew abundantly, the bees had incorporated with the honey sufficient poison from these flowers to account for numerous and occasional fatal cases of poisoning.—*Med. and Surg. Reporter.*

---

A quack doctor in Pennsylvania, in answer to the shrewd questions of deluding counsel, recently testified in court that he decapitated his patient, the defendant in the case, performed a Cæsarean section upon him, and finally made an autopsy and cured him. It was not the quack's regular day for lying, either.

---

The Archives of Gynæcology, Obstetrics and Pædiatrics, New York, series of 1886, just completed, has met with such warm encouragement that the publishers have decided to issue it monthly, and commencing with January the parts will so appear, instead of bi-monthly as heretofore.

---

"Good morning, Mrs. Gilligan ; how is Patrick this morning ?" "Sure, he's no better, sir." "Why don't you send him to the hospital to be treated ?" "To be treated, is it ? Faith an' it's the delarium trimmins he has already."

---

Swift once said that the reason a certain university was a learned institution was that most persons took a little learning there, and few brought any away with them, and so the learning accumulated.

---

What is more pathetic than to see the simple faith with which a bald-headed customer will buy an infallible hair restorer from a bald-headed barber ?

